# Spatio-Temporal Mutational Profile Appearances of Swedish SARS-CoV-2 during the Early Pandemic

**DOI:** 10.3390/v12091026

**Published:** 2020-09-14

**Authors:** Jiaxin Ling, Rachel A. Hickman, Jinlin Li, Xi Lu, Johanna F. Lindahl, Åke Lundkvist, Josef D. Järhult

**Affiliations:** 1 Department of Medical Biochemistry and Microbiology, Zoonosis Science Center, University of Uppsala, SE-751 23 Uppsala, Sweden; lijinlinwhu@gmail.com (J.L.); Johanna.Lindahl@imbim.uu.se (J.F.L.); ake.lundkvist@imbim.uu.se (Å.L.); 2 Department of Cell and Molecular Biology, Karolinska Institutet, SE-171 77 Stockholm, Sweden; 3 School of Life Sciences, Sun Yat-sen University, Guangzhou 510275, China; shindowfeather@gmail.com; 4 International Livestock Research Institute, Nairobi 00100, Kenya; 5 Swedish University of Agricultural Research, SE-750 07 Uppsala, Sweden; 6 Zoonosis Science Center, Department of Medical Sciences, Uppsala University, SE-751 23 Uppsala, Sweden; josef.jarhult@medsci.uu.se

**Keywords:** SARS-CoV-2, evolution, mutation, spike protein

## Abstract

Background: During the COVID-19 pandemic, the virus evolved, and we therefore aimed to provide an insight into which genetic variants were enriched, and how they spread in Sweden. Methods: We analyzed 348 Swedish SARS-CoV-2 sequences freely available from GISAID obtained from 7 February 2020 until 14 May 2020. Results: We identified 14 variant sites ≥5% frequency in the population. Among those sites, the D936Y substitution in the viral Spike protein was under positive selection. The variant sites can distinguish 11 mutational profiles in Sweden. Nine of the profiles appeared in Stockholm in March 2020. Mutational profiles 3 (B.1.1) and 6 (B.1), which contain the D936Y mutation, became the predominant profiles over time, spreading from Stockholm to other Swedish regions during April and the beginning of May. Furthermore, Bayesian phylogenetic analysis indicated that SARS-CoV-2 could have emerged in Sweden on 27 December 2019, and community transmission started on February 1st with an evolutionary rate of 1.5425 × 10^−3^ substitutions per year. Conclusions: Our study provides novel knowledge on the spatio-temporal dynamics of Swedish SARS-CoV-2 variants during the early pandemic. Characterization of these viral variants can provide precious insights on viral pathogenesis and can be valuable for diagnostic and drug development approaches.

## 1. Introduction

A new pandemic, coronavirus disease 2019 (COVID-19), emerged in 2019 and was caused by a new coronavirus, designated as severe acute respiratory syndrome coronavirus-2 (SARS-CoV-2) [1,2,3]. SARS-CoV-2 is a positive-sense single-stranded RNA virus with a genome of about 30 kb that encodes four structural proteins: the spike (S) protein, the envelope (E) protein, the matrix (M) protein, and the nucleocapsid (N) protein; together with 8 accessory proteins and 16 non-structural proteins [4], including the RNA-dependent RNA polymerase (RdRp) and the nsP14 with exonuclease activity and proof-reading function [5]. Due to its high transmissibility and lack of pre-existing immunity against this novel virus in the human population, the rapid spread of SARS-CoV-2 is currently a huge threat to public health and global economies.

The first case of COVID-19 in Sweden was reported on 31 January 2020 from a woman returning from Wuhan to Jönköping. Soon after that, several introductions of COVID-19 cases were reported and all were travel-related cases from Italy and Iran. The community transmission of COVID-19 was thought to start in late February, especially in the Stockholm, Sörmland, Uppsala, Västra Götaland, Örebro, and Östergötland regions. During the early pandemic (until 14 May), a total of 29,739 COVID-19 cases and 3834 deaths had been confirmed in Sweden [6].

To prevent the spread of SARS-CoV-2, many countries adopted strict non-pharmacological interventions (NPIs) such as lockdowns, travel restrictions, and widespread business and school closures to stop the transmission [7]. However, in response to the pandemic, Sweden took a unique strategy where less strict NPIs were implemented. Instead, social recommendations were advised, with the aim to slow down the spread of the virus and protect the risk group at the same time [8]. This unique strategy was meant to slow down the viral transmission in the population, but as compared to more strict strategies, resulted in a higher probability of a slow but continuous evolution of the virus. The high level of transmission provided us with an opportunity to investigate the evolutionary profiling of Swedish SARS-CoV-2 over time.

This descriptive study is based on Swedish SARS-CoV-2 sequences that are freely available from GISAID [9], where we compared how these strains diverged from the Wuhan prototype of SARS-CoV-2. The study traced the dynamic mutational profiles of SARS-CoV-2 in Sweden and calculated the time points when community transmission for these mutational profiles likely started. These viral characteristics help us to understand how SARS-CoV-2 spread under the current Swedish mandates against COVID-19 and the evolutionary traits it acquired within this time-frame.

## 2. Materials and Methods

### 2.1. Data Sets

The multiple alignment sequences and metadata including the sequence information, locations, and collection date were downloaded from GISAID (https://www.gisaid.org/) on 9 June 2020. The Swedish data was pulled out from the global dataset that contains 38,139 sequences. These 354 sequences were originally sequenced by the Swedish Public Health Agency (FHM) and Centre for Translation Microbiome Research (CTMR) at the Karolinska Institutet. Six sequences were excluded due to lack of information on sampling date. The remaining 348 Swedish sequences and metadata were included into the downstream analysis. Together with the reference sequence (NC, 045512), a total of 349 sequences were realigned by using MAFFT with default settings, followed by manual refinement using Geneious prime (https://www.geneious.com/prime/) [10]. The metadata (Table S1) was imported into R studio for data visualization [11].

### 2.2. Mutation Sites in Swedish SARS-CoV-2 Sequences

The sequence dataset (348 Swedish SARS-CoV-2 sequences) and the reference sequence (Severe acute respiratory syndrome coronavirus 2 Wuhan-Hu-1, GenBank accession number. NC, 045512) [5] were imported into Geneious prime and the alignment was searched for variants/SNPs. A minimum variant frequency of 0.05 was used as the cut-off with the default settings for *p*-value testing, which is recommended by Geneious prime and in other genetic population studies [12,13]. The variant frequency, locations, mutation type, and the nature of amino acid (aa) mutations in the population of Swedish SASR-CoV-2 were analyzed.

### 2.3. Recombination Analysis and Selection Pressure

Potential recombination events were investigated using RDP3 [14]. The selection pressure for each gene and branches were analysed using the following methods: MEME (Mixed Evolutionary Model of Evolution), FEL (Fixed Impact Probability), FUBAR (Fast Unconstrained Bayesian AppRoximation), and aBSREL (adaptive branch-site REL test for episodic diversification) implemented in Datamonkey (https://www.datamonkey.org) [15].

### 2.4. Evolutionary Dynamics Analysis of Mutational Profiles

A mutational profile for each sequence was created from all the variants detected with ≥0.05 cut-off, where all variants/mutations were concatenated together to make a mutational profile. Within our sequence data-set, we observed 11 different mutational profiles (Table S1). Data was imported into R studio for visualization using the packages ggplot2 and ggmuller to obtain a Muller plot of the Swedish variants and the longitudinal cumulative mutational profile frequency [11]. Further data visualization of the metadata of mutational profiles 3 and 6 was done by importing the data into Spyder using the python packages matplotlib, numpy, and seaborn (https://www.python.org/download/releases/3.0/).

### 2.5. Phylogenetic Inference

RAxML was used to reconstruct the phylogenetic relationship of Swedish SARS-CoV-2 and the other variants globally [16]. To reconstruct the evolutionary history of the Swedish SARS-CoV-2, Bayesian phylogenetic trees of the complete sequences were constructed by employing BEAST v1.8.4. Bayesian analysis consisted at least 50 million Bayesian Monte Carlo Markov chain (MCMC) generations sampling every 5000 generations. The run was continued until convergence was obtained (average deviation, <0.01) and with a 25% burn-in. To further infer the evolutionary rates and the most recent common ancestor (tMRCA) of the mutational profiles, we first employed TempEst to test if the dataset had a clocklike structure [17]. A regression of root-to-tip genetic distances of the dataset (348 Swedish sequences and one from Wuhan) against date of sampling showed a clocklike structure (correlation coefficient, 0.4736). Consequently, we used 6 different combinations of demographic and molecular clock models and ran 50 million Bayesian MCMC generations sampling every 5000 generations, implemented in BEAST v1.8.4 (Table S2). Model comparison was performed by a marginal-likelihood estimator in two approaches, path sampling (PS) and stepping-stone sampling (SS); and selected strict clock and exponential population as a better model for data analysis, with the log Bayesian factor (BF) value over at least 25. In all analyses, the prototype (GenBank accession number NC, 045512), was used to root the tree. All computations were run using the CIPRES computational cluster (http://www.phylo.org/index.php/). Finally, trees and sequence ID with the information of mutational profiles were viewed and edited using FigTree v1.4.2 (http://tree.bio.ed.ac.uk/software/figtree/).

## 3. Results

### 3.1. Sequence Collection Overview

Our dataset (Table S1) comprises Swedish SARS-CoV-2 sequences from samples obtained between 7 February 2020 and 14 May 2020 (the first 98 days of the pandemic in Sweden). The age distribution of the study consisted of 60 sequences obtained from COVID-19 patients that were ≥60 years old, 281 sequences obtained from COVID-19 patients ≤59 years old, and 7 with unknown age. There was a slight gender bias of 179 sequences from men, 164 from women, and 5 with undisclosed gender metadata. The sequences included in this study were from 18 different geographical locations across Sweden, with the largest number of 149 sequences obtained from Stockholm, the capital of Sweden.

There were no individual sites identified as episodic positive/diversifying selection by MEME, FEL, and aBSREL. However, the FUBAR test showed that four sites (L3606F and G392D on ORF1ab, D936Y on S protein, T205I on N protein) were under episodic positive/diversifying selection with posterior probability ≥ 0.95 and log BF ≥ 25. There were no recombination events observed.

### 3.2. Divergence of SARS-CoV-2 in Sweden as Compared to the Prototype Sequence from Wuhan, China

We used a 5% minimum frequency as an arbitrary cut-off for searching the variants for the Swedish SARS-CoV-2 variants. Table 1 shows all 14 mutational sites and their frequencies observed in the dataset. From these 14 mutational sites, 2 were synonymous, 11 were non- synonymous, and 1 occurred in a non-coding region. Seven out of the 11 non-synonymous mutations also conferred functional modification to coding amino acid group, such as a negatively charged R group to a non-polar aliphatic R group. The main mutational driving force seems to be transitional single nucleotide mutations, with C → T being the most prevalent. This could be a result of c-deamination, which is ubiquitous in nature.

### 3.3. Mutational Profiles of Swedish SARS-CoV-2

In Sweden, a total of 11 mutational profiles (MPs) were circulating during the early pandemic (Table 2). Mutations C241T, C3037T, C14408T, and A23403G (MP4) provided the basis for the other MPs patterns; meanwhile, mutations G28881A, G28882A, and G28883C appeared together. Combination of these two mutation patterns constituted MP3. The basis, together with mutations C1059T, G24368T, and G25563T, constituted MP6. Different MPs or mutational combinations might be beneficial for viral evolution.

**Figure 1:**
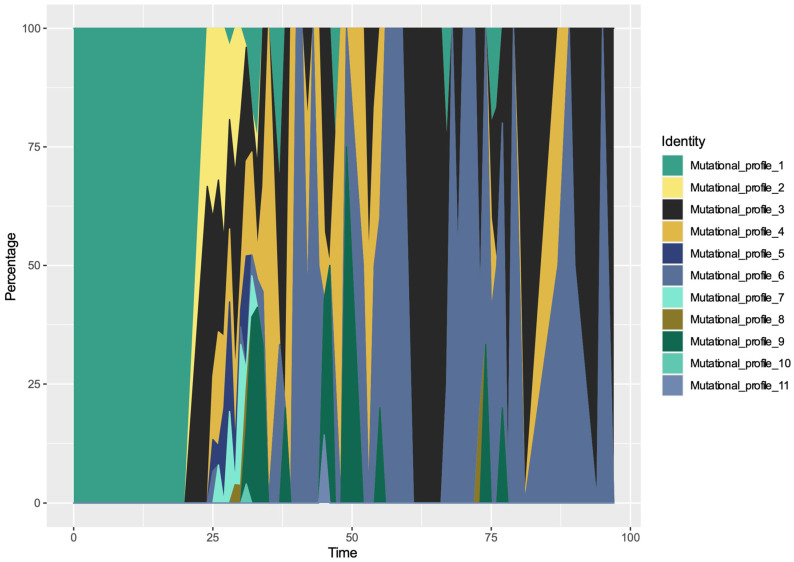
Population dynamics of Swedish SARS-CoV-2 mutational profile over the time. A Muller plot revealed 11 mutational profiles of SARS-CoV-2 in Sweden.

From Figure 1, we can observe the introduction of SARS-CoV-2 virus into Sweden from international sources with occasional re-introductions. As expected, the very early mutational profile (MP1) constituted travel-associated cases, which were the main Swedish COVID-19 cases in February. From the beginning of March, local transmission was the main driver for SARS-CoV-2 in Sweden, i.e., 10 other MPs in addition to MP1 had emerged in the Swedish population. A Muller plot depicts the mutational profile dynamics during these 98 days (Figure 1). MP 3 and MP 6 were established as the dominant mutational profiles over our study time-period. On our last time-point, MP3 was most prevalent in the dataset.

### 3.4. Spatio-Temporal Mutational Profile Appearances in Swedish SARS-CoV-2 Variants

To further investigate the spatial-temporal appearances of these 11 mutational profiles, we plotted the accumulated counts for each profile by months and locations (Figure S1). As observed in the Muller plot, MP3 and MP6 had been outcompeting the other MPs and became the dominant MPs in Sweden, followed by MP4, MP9, and MP1. MP6 increased significantly after day 40 (March 17) and reached the second highest of the accumulated mutant accounts. The facet grid multiple plots showed that in March, nine MPs (all except MP7 and MP11) were circulating in Stockholm. In contrast, MP7 originated in Värmland, and MP11 originated in Halland. In April, although very few sequences represented Stockholm due to limited sampling, MP3, MP4, and MP6 were still disseminated in different regions in Sweden, especially in Uppsala and Västra Götaland (Figure S1).

**Figure 2:**
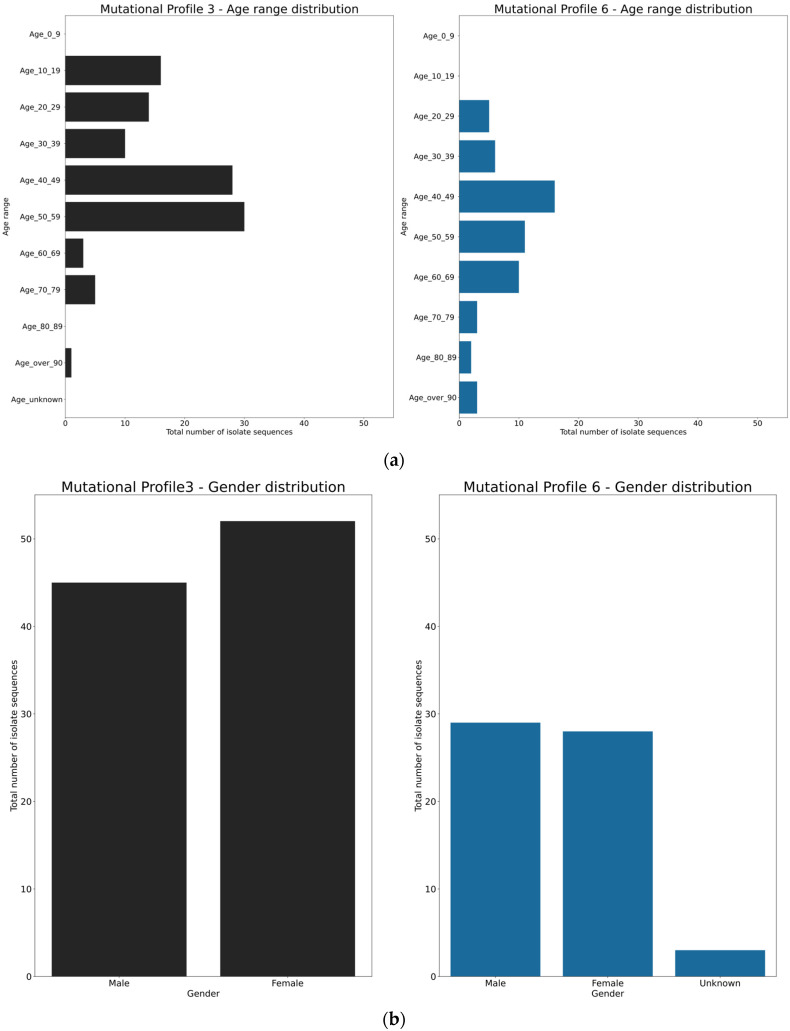

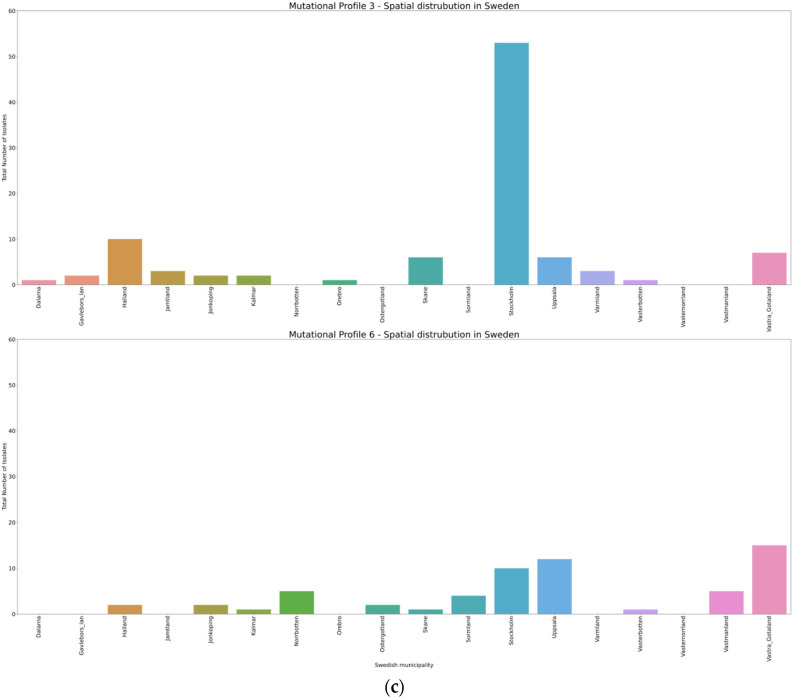
Demographic characterizations of mutational profiles 3 and 6 by age (**a**), gender (**b**), and geographical location (**c**).

Further analyses on spatial and infected individual characteristics were made for MP3 and MP6. The GISAID contains limited metadata for the Swedish sequences, with the maximal information being patient age, gender, geographic location, and date that the sample was collected. From these limited data, we compiled metadata plots on MP3 and MP6 (Figure 2). It appears that the age group from 40 to 49 years was the target group for both MP3 and MP6, whilst MP3 also had a preference for the age group of 10 to 19 years, with a gender bias and distributed in the geographic locations with the order of highest number in Stockholm (Figure 2).

### 3.5. Bayesian Phylogenetic Analysis

**Figure 3:**
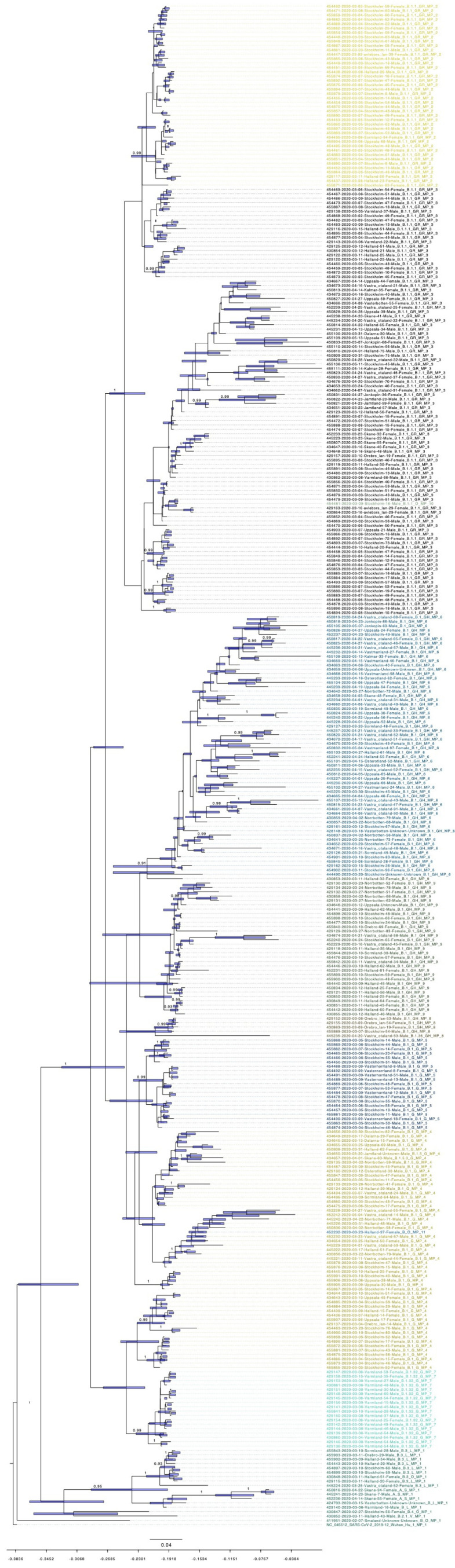
Dated phylogenetic tree of Swedish SARS-CoV-2. Posterior probability values are shown on the branches. The bar on the nodes show the confidence of the time with 95% highest posterior density (HPD). The time scale on the bottom indicates time before 14 May 2020, and tick values on the scale represent every 14 days. The color indicator is the same as in Figure 1.

Model comparison preferred a strict molecular clock mode and a coalescent exponential population demographic model for the evolutionary history of Swedish SARS-CoV-2 (Figure 3). The evolutionary rate for this dataset is 1.5425 × 10^−3^ substitutions per year (95% highest posterior density (HPD), 1.2795 × 10^−3^ to 1.8259 × 10^−3^; Table S2.). Rapid community transmission started on the 1 February (95% HPD, 16 January to 14 February). The emergence of MP3 and MP6 were on 17 February (95% HPD, 8 February to 24 February) and 25 February (95% HPD, 14 February to 9 March), respectively. Phylogenetic analyses showed that almost all different MPs cluster together, except MP10 that fell into the MP3 cluster, and MP11 that fell into the MP4 cluster. MP4, MP5, MP6, MP8, and MP9 belong to lineage B.1, while MP2 and MP3 belong to B.1.1.

## 4. Discussion

Continuous molecular tracing of SARS-CoV-2 is needed for effective surveillance and interventions. FHM has been monitoring the molecular traits of Swedish SARS-CoV-2 since the initial cases in Sweden. Two reports from FHM, not yet published in peer-reviewed journals but available on their web-page (www.folkhalsomyndigheten.se), indicate that the initial introductions of SARS-CoV-2 to Sweden originate from Italy and Austria [18,19]. Our study has similar findings to the FHM reports with independent genotypes circulating, which are highly likely to have originated from independent geographic locations. However, our study is more focused on the genetic variations among the Swedish SARS-CoV-2 sequences and the evolutionary events that have occurred.

Most RNA virus populations exist as complex mixtures of genetic and phenotypic variants, resulting from the high RNA polymerase error rate [20]. The theoretical advantage of maintaining such a diverse viral population is that a variant might fit into a new environment when the virus spreads. In certain circumstances, some mutations could be drivers for the emergence of new trains with changed pathogenicity. For instance, a mutation in the Zika virus membrane region (prM-S139N) emerged in a viral lineage preceding the devastating epidemic in the Americas [21], while a single mutation (GP-A82V) in Ebola virus increased the infection rate of human cells [22]. However, coronaviruses have RDRp and nsP14 proteins with proofreading, and therefore mutations occur at a lower rate as compared to most other RNA viruses [23]. Still, genetic drift is the main evolutionary mode for Swedish SARS-CoV-2, and the wide spreading of SARS-CoV-2 have already resulted in different clades/lineages that differ from the original strain from Wuhan, where the first cases were found (Figure S1). There is no information available on whether these variants could affect the transmissibility or infectivity of SARS-CoV-2. The continuous pandemic may enable accumulation of immunologically relevant mutations in the SARS-CoV-2 genome [24]. Point mutations have been shown to result in resistance to neutralizing antibodies in MERS-CoV [25] and SARS-CoV [26]. Antigenic drift has been demonstrated in other CoVs, including the common cold coronaviruses OC43 and 229E, and SARS-CoV [27,28,29,30]. Our findings that D936Y in the S protein is under positive selection is consistent with antigenic drift playing a role for SARS-CoV-2 as well. The S protein of SARS-CoV-2 is responsible for viral entry into host cells through the receptor binding domain (RBD). Mutations in the S protein may impact development of pharmacological interventions and sensitive diagnostic methods. However, the functional change of this mutation is still unclear. One study using mutant modelling and analysis showed that it could weaken the post-fusion assembly for the virus [31]. Although the frequency of S936Y is low worldwide, increased frequency has been observed in Nordic countries: 69% (178/258, the number for mutant’s appearance/total number of SARS-CoV-2) in Finland, 22% (116/531) in Sweden, and 11% (9/83) in Norway (data from 3 August, http://covid19.datamonkey.org).

Our study also indicates that SARS-CoV-2 evolves through certain mutational profiles, i.e., multiple genes are likely involved in the evolution. A mutated virus must contain multiple mutations in different genes in order to keep up with stringent evolutionary constraints [32]. Those mutations that are favoured by natural selection can spread in the population and act as the mutational backbone for further genetic variants to evolve from. For our study, we set a ≥5% frequency threshold in the population as the cut-off for the variant sites. We found that the basis mutations, which contain C241T, C3037T, C14408T, and A23403G, combined with other mutations can be classified into 10 mutational profiles in Sweden. A23403G is one of the most prominent mutations; it occurs in the S protein at amino acid residue 614, where Aspartic acid is substituted by Glycine (D614G). The D614G mutant strain is designated as the “G clade” by GISAID and originated in Europe, and further spread to North America and Oceania, then Asia [33]. This mutation can increase infectivity of SARS-CoV-2 based on in vitro experiments [24]. In Sweden, we found that on 14 May, the frequency of D614G on the S1 protein was 94.8% in the population. All MPs with the exception of MP1 had the basic genomic mutation A23403G. Out of the 10 mutational profiles, MP6 appeared latest within our investigation period and could have the carrying capacity to outcompete MPs in the population after our time-frame. Cavallo L. et al. found that the D614G/ D936Y co-occur on the S1/S2 protein, and their emergence was traced back to 15 March in Washington, USA, and later on spread to Wales, Iceland, and the Netherlands [31]. This provides more evidence that multiple mutations can modulate viral transmission, replication efficiency, and virulence in different regions of the world [34]. Therefore, exploring mutational profiles of sequences is an important complement to analysing single nucleotide polymorphisms and may be more efficient. We saw this co-occurrence of D614G/D936Y in our data-set with a frequency of 17.2%, which was the same frequency as MP6. MP6 has the same mutations as in the findings of Cavallo L. et al., but with the additional mutations T265I on ORF1ab, Q57H on ORF3, and the four basic mutations (C241T, C3037T, C14408T, and A23403G). We are unable to ascertain the function of the additional mutations found in MP6 compared to S1/S2 protein findings: this will require additional characterization.

Due to high viral transmissibility and lack of pre-existing immunity, COVID-19 cases surged in late February and March, mainly in Stockholm. From our Bayesian phylogenetic method, we have calculated the emergence of COVID-19 in Sweden and the start of community transmission, which occurred in Stockholm. We found 1.5425 × 10^−3^ substitutions per year as the evolutionary rate of Swedish SARS-CoV-2 by using the formal Bayesian inference. This is similar to earlier reports that demonstrated 1.12 × 10^−3^ substitutions per year for SARS-CoV-2 [35,36]. However, substitution rates may be overestimated, as most mutations are under purifying selection [37]. In addition, this analysis requires caution due to some uncertainties as a result of small sampling size and model selections during the estimations. Therefore epidemiological evidences have to be incorporated to the analysis, to reduce the descriptive conclusions of this study [38]. During the pandemic, there have been frequent updates for new sequenced isolates with evolving nomenclature systems for SARS-CoV-2 such as Nextstrain, GISAID, and PANGOLIN. According to the PANGOLIN system [39], lineage B.1 is the predominant global lineage, which comprises the large Italian outbreak and is also associated with many outbreaks in Europe [40]. Lineage B.1.1 is the main lineage in Europe and was exported to several areas of the world [39]. B.1 and B.1.1 are the major lineages in Sweden. To further see if how these major lineages transmitted into Sweden, the report from FMH compared the single nucleotide polymorphism (SNP) profiles of Swedish sequences and the sequences from Italy and Austria within the B.1 and B.1.1. They found a clear link between the sequences from Sweden and Italy within B.1.1. They also observed similarities between sequences from Sweden and Austria within B.1. However, unlike the Swedish B.1 isolates, the Austrian sequences had no mutations in the S protein at position 936. One explanation of the result seen by FHM could be that further mutational evolution occurred in Sweden or another geographical location, or that not enough sequencing in Austria was done to detect these mutations. Unlike the FMH reports, our mutational profiles systems, on the other hand, can further distinguish those genetic variances with more precision, as B.1 can be further divided into MP4, MP5, MP6, MP8, and MP9, while B.1.1 can be further divided into MP2, MP3, and MP10. This additional information can aid in the assessment of the evolutionary paths that SARS-CoV-2 virus can take to become the predominant genotypes in the population. From remapping the mutational profiles involved in our analysis in Figure 3 and Table 2, we can see a clear clustering pattern that still matches with the PANGOLIN and GISAID classification systems that standardized SARS-CoV-2 nomenclature. Therefore, the use of mutation profiles can be used in conjunction with other SARS-CoV-2 nomenclature systems to aid in showing the local sub-populations that occur in a given location during the SARS-CoV-2 pandemic, such as those presented in our paper.

## 5. Conclusions

Further molecular surveillance on Swedish SARS-CoV-2 is needed to determine whether the two mutation patterns MP3 (B.1.1) and MP6 (B.1) will be fixed over time. Importantly, characterizing viruses with these two major mutational profiles in greater depth may aid in understanding viral infectivity and transmissibility, and potentially add further treatment prospects for COVID-19 patients in Sweden and worldwide. Mutational profiling may be an efficient additional tool for SARS-CoV-2 molecular epidemiology within a geographical location.

## Figures and Tables

**Table 1:** Observed mutations and the frequency.

Site (NC, 045512)	Ref (NC, 045512)	Variant Change	Wild Type Frequency (%)	Variant Frequency (%)	aa Change	Type	Region	Amino Acid Property Change
241	C	T	5.2	94.8		transition	5′UTR gene	
1059	C	T	73.6	26.4	T265I	transition	ORF1ab/nsp2	Polar, uncharged R group to Non-polar aliphatic R group
3037	C	T	5.7	94.3	F924F	transition	ORF1ab/nsp3	
12790	A	G	94.8	5.2	T4175T	transition	ORF1ab/nsp9	
13568	C	T	94.8	5.2	A43V	transition	RNA-dependent RNA polymerase	Both are non-polar aliphatic R groups
14408	C	T	6.0	94.0	P323L	transition	RNA-dependent RNA polymerase	Polar, uncharged R group to Non-polar aliphatic R group
22583	G	A	94.8	5.2	V341I	transition	S	Both are non-polar aliphatic R groups
23403	A	G	5.2	94.8	D614G	transition	S	Negatively charged R group to Non-polar aliphatic R group
24368	G	T	82.8	17.2	D936Y	transversion	S	Negatively charged R group to Polar aliphatic R group
25563	G	T	72.2	27.8	Q57H	transversion	ORF3a	Negatively charged R group to Positively charged R group
27046	C	T	88.0	12.0	T175M	transition	M	Polar, uncharged R group to Non-polar aliphatic R group
28881	G	A	59.9	40.1	R203K	transition	N	Both are positive R groups
28882	G	A	59.9	40.1	R203K	transition	N	Both are positive R groups
28883	G	C	59.9	40.1	G204R	transversion	N	Non-polar aliphatic R groups to Positively charged R group

**Table 2:** Mutational profiles observed in Swedish sequences.

Profiles	PANGOLIN Lineage	GISAID Lineage	Frequency (%)	Mutational Profiling
Mutational Profile 1	B	O	4.89	WT
	B.4	O		WT
	B	L		WT
	B.3	L		WT
	B.3	O		WT
	B.2.1	V		WT
	A	S		WT
Mutational Profile 2	B.1.1	GR	12.1	241C > T, 3037C > T, 14408C > T, 23403A > G, 27046C > T, 28881G > A, 28882G > A, 28883G > C
Mutational profile 3	B.1.1	GR	27.9	241C > T, 3037C > T, 14408C > T, 23403A > G, 28881G > A, 28882G > A, 28883G > C
	B.1.1.1	GR		241C > T, 3037C > T, 14408C > T, 23403A > G, 28881G > A, 28882G > A, 28883G > C
Mutational profile 4	B.1	G	15.5	241C > T, 3037C > T, 14408C > T, 23403A > G
	B.1.5	G		241C > T, 3037C > T, 14408C > T, 23403A > G
	B.1.5.3	G		241C > T, 3037C > T, 14408C > T, 23403A > G
Mutational Profile 5	B.1	G	6.03	241C > T, 3037C > T, 14408C > T, 22583G > A, 23403A > G
Mutational Profile, 6	B.1	GH	17.2	241C > T, 1059C > T, 3037C > T, 14408C > T, 23403A > G, 24368G > T, 25563G > T
Mutational Profile 7	B.1.32	G	5.17	241C > T, 3037C > T, 12790A > G, 14408C > T, 14408C > T, 23403A > G
Mutational Profile 8	B.1	GH	1.43	241C > T, 3037C > T, 14408C > T, 23403A > G, 25563G > T
Mutational Profile, 8	B.1.36	GH		241C > T, 3037C > T, 14408C > T, 23403A > G, 25563G > T
Mutational Profile 9	B.1	GH	9.20	241C > T, 1059C > T, 3037C > T, 14408C > T, 23403A > G, 25563G > T
Mutational Profile 10	B.1.1	O	0.287	241C > T, 14408C > T, 23403A > G, 28881G > A, 28882G > A, 28883G > C
Mutational Profile 11	B	O	0.287	241C > T, 14408C > T, 23403A > G

## Supplementary Materials

The following are available online at https://www.mdpi.com/1999-4915/12/9/1026/s1. Figure S1. (a) Longitudinal cumulative mutational profile frequency of the sequenced Swedish strains. (b) Mutational profile preference indicated by months and locations. Figure S2. Maximum-likelihood phylogenetic tree of SARS-CoV-2. The taxa color in red represents the variants from Wuhan and blue are from Sweden. Table S1. Spatial temporal appearance of each variant and their mutation profile and clade information. Table S2. Model comparison by BEAST analysis.

## References

[1] Guarner J. (2020). Three Emerging Coronaviruses in Two Decades. Am. J. Clin. Pathol..

[2] De Wit E., van Doremalen N., Falzarano D., Munster V.J. (2016). SARS and MERS: Recent insights into emerging coronaviruses. Nat. Rev. Microbiol..

[3] Hilgenfeld R., Peiris M. (2013). From SARS to MERS: 10 years of research on highly pathogenic human coronaviruses. Antivir. Res..

[4] Wu A., Peng Y., Huang B., Ding X., Wang X., Niu P., Meng J., Zhu Z., Zhang Z., Wang J. (2020). Genome Composition and Divergence of the Novel Coronavirus (2019-nCoV) Originating in China. Cell Host Microbe.

[5] Wu F., Zhao S., Yu B., Chen Y.M., Wang W., Song Z.G., Hu Y., Tao Z.W., Tian J.H., Pei Y.Y. (2020). A new coronavirus associated with human respiratory disease in China. Nature.

[6] Folkhälsomyndigheten. https://www.folkhalsomyndigheten.se.

[7] Walker P.G.T., Whittaker C., Watson O.J., Baguelin M., Winskill P., Hamlet A., Djafaara B.A., Cucunuba Z., Mesa D.O., Green W. (2020). The impact of COVID-19 and strategies for mitigation and suppression in low- and middle-income countries. Science.

[8] Kamerlin S.C.L., Kasson P.M. (2020). Managing COVID-19 spread with voluntary public-health measures: Sweden as a case study for pandemic control. Clin. Infect. Dis..

[9] Elbe S., Buckland-Merrett G. (2017). Data, disease and diplomacy: GISAID’s innovative contribution to global health. Glob. Chall..

[10] Katoh K., Standley D.M. (2013). MAFFT multiple sequence alignment software version 7: Improvements in performance and usability. Mol. Biol. Evol..

[11] Team R.C. (2020). R: A Language and Environment for Statistical Computing.

[12] Wang G.T., Peng B., Leal S.M. (2014). Variant association tools for quality control and analysis of large-scale sequence and genotyping array data. Am. J. Hum. Genet..

[13] Speed D., Hemani G., Johnson M.R., Balding D.J. (2012). Improved heritability estimation from genome-wide SNPs. Am. J. Hum. Genet..

[14] Martin D.P., Lemey P., Lott M., Moulton V., Posada D., Lefeuvre P. (2010). RDP3: A flexible and fast computer program for analyzing recombination. Bioinformatics.

[15] Weaver S., Shank S.D., Spielman S.J., Li M., Muse S.V., Pond S.L.K. (2018). Datamonkey 2.0: A Modern Web Application for Characterizing Selective and Other Evolutionary Processes. Mol. Biol. Evol..

[16] Stamatakis A. (2014). RAxML version 8: A tool for phylogenetic analysis and post-analysis of large phylogenies. Bioinformatics.

[17] Rambaut A., Lam T.T., Carvalho L.M., Pybus O.G. (2016). Exploring the temporal structure of heterochronous sequences using TempEst (formerly Path-O-Gen). Virus Evol..

[18] Folkhälsomyndigheten (2020). Helgenomsekvensering av Svenska SARS-CoV-2 som Orsakar COVID-19. https://www.folkhalsomyndigheten.se/publicerat-material/publikationsarkiv/h/helgenomsekvensering-av-svenska-sars-cov-2-som-orsakar-covid-19/.

[19] Folkhälsomyndigheten (2020). Helgenomsekvensering av Svenska SARS-CoV-2 som Orsakar COVID-19. https://www.folkhalsomyndigheten.se/publicerat-material/publikationsarkiv/h/helgenomsekvensering-av-svenska-sars-cov-2-som-orsakar-covid-19-del2/.

[20] Lauring A.S., Andino R. (2010). Quasispecies theory and the behavior of RNA viruses. PLoS Pathog..

[21] Yuan L., Huang X.Y., Liu Z.Y., Zhang F., Zhu X.L., Yu J.Y., Ji X., Xu Y.P., Li G., Li C. (2017). A single mutation in the prM protein of Zika virus contributes to fetal microcephaly. Science.

[22] Urbanowicz R.A., McClure C.P., Sakuntabhai A., Sall A.A., Kobinger G., Muller M.A., Holmes E.C., Rey F.A., Simon-Loriere E., Ball J.K. (2016). Human Adaptation of Ebola Virus during the West African Outbreak. Cell.

[23] Cyranoski D. (2020). Profile of a killer: The complex biology powering the coronavirus pandemic. Nature.

[24] Korber B.F., Fischer W.M., Gnanakaran S., Yoon H., Theiler J., Abfalterer W., Hengartner N., Giorgi E.E., Bhattacharya T., Foley B. (2020). Tracking changes in SARS-CoV-2 Spike: Evidence that D614G increases infectivity of the COVID-19 virus. Cell.

[25] Tang X.C., Agnihothram S.S., Jiao Y., Stanhope J., Graham R.L., Peterson E.C., Avnir Y., Tallarico A.S., Sheehan J., Zhu Q. (2014). Identification of human neutralizing antibodies against MERS-CoV and their role in virus adaptive evolution. Proc. Natl. Acad. Sci. USA.

[26] Sui J., Li W., Roberts A., Matthews L.J., Murakami A., Vogel L., Wong S.K., Subbarao K., Farzan M., Marasco W.A. (2005). Evaluation of human monoclonal antibody 80R for immunoprophylaxis of severe acute respiratory syndrome by an animal study, epitope mapping, and analysis of spike variants. J. Virol..

[27] Ren L., Zhang Y., Li J., Xiao Y., Zhang J., Wang Y., Chen L., Paranhos-Baccala G., Wang J. (2015). Genetic drift of human coronavirus OC43 spike gene during adaptive evolution. Sci. Rep..

[28] Vijgen L., Keyaerts E., Lemey P., Moes E., Li S., Vandamme A.M., Van Ranst M. (2005). Circulation of genetically distinct contemporary human coronavirus OC43 strains. Virology.

[29] Chibo D., Birch C. (2006). Analysis of human coronavirus 229E spike and nucleoprotein genes demonstrates genetic drift between chronologically distinct strains. J. Gen. Virol..

[30] Guan Y., Zheng B.J., He Y.Q., Liu X.L., Zhuang Z.X., Cheung C.L., Luo S.W., Li P.H., Zhang L.J., Guan Y.J. (2003). Isolation and characterization of viruses related to the SARS coronavirus from animals in southern China. Science.

[31] Cavallo L., Oliva R. (2020). D936Y and Other Mutations in the Fusion Core of the SARS-Cov-2 Spike Protein Heptad Repeat 1 Undermine the Post-Fusion Assembly. bioRxiv.

[32] Grubaugh N.D., Petrone M.E., Holmes E.C. (2020). We shouldn’t worry when a virus mutates during disease outbreaks. Nat. Microbiol..

[33] Phan T. (2020). Genetic diversity and evolution of SARS-CoV-2. Infect. Genet. Evol..

[34] Pachetti M., Marini B., Benedetti F., Giudici F., Mauro E., Storici P., Masciovecchio C., Angeletti S., Ciccozzi M., Gallo R.C. (2020). Emerging SARS-CoV-2 mutation hot spots include a novel RNA-dependent-RNA polymerase variant. J. Transl. Med..

[35] Boni M.F., Lemey P., Jiang X., Lam T.T.-Y., Perry B.W., Castoe T.A., Rambaut A., Robertson D.L. (2020). Evolutionary origins of the SARS-CoV-2 sarbecovirus lineage responsible for the COVID-19 pandemic. Nat. Microbiol..

[36] Koyama T., Daniel P., Parida L. (2020). Variant analysis of SARS-CoV-2 genomes. Bull. World Health Organ..

[37] Membrebe J.V., Suchard M.A., Rambaut A., Baele G., Lemey P. (2019). Bayesian Inference of Evolutionary Histories under Time-Dependent Substitution Rates. Mol. Biol. Evol..

[38] Warnock R.C., Yang Z., Donoghue P.C. (2012). Exploring uncertainty in the calibration of the molecular clock. Biol. Lett..

[39] Rambaut A., Holmes E.C., O’Toole A., Hill V., McCrone J.T., Ruis C., du Plessis L., Pybus O.G. (2020). A dynamic nomenclature proposal for SARS-CoV-2 lineages to assist genomic epidemiology. Nat. Microbiol..

[40] Lai A., Bergna A., Caucci S., Clementi N., Vicenti I., Dragoni F., Cattelan A.M., Menzo S., Pan A., Callegaro A. (2020). Molecular Tracing of SARS-CoV-2 in Italy in the First Three Months of the Epidemic. Viruses.

