# Supplementary material for: Spatio-Temporal Mutational Profile Appearances of Swedish SARS-CoV-2 during the Early Pandemic
Source: Viruses. 2020 Sep 14;12(9):1026. doi: 10.3390/v12091026 (PMC7551444; doi:10.3390/v12091026)
Supplement: Supplementary file 1 [file viruses-12-01026-s001.zip › Figure S1_200913.docx]

**Figure S1. (a)** Longitudinal cumulative mutational profile frequency of the sequenced Swedish strains. **(b)** Mutational profile preference indicated by months and locations.


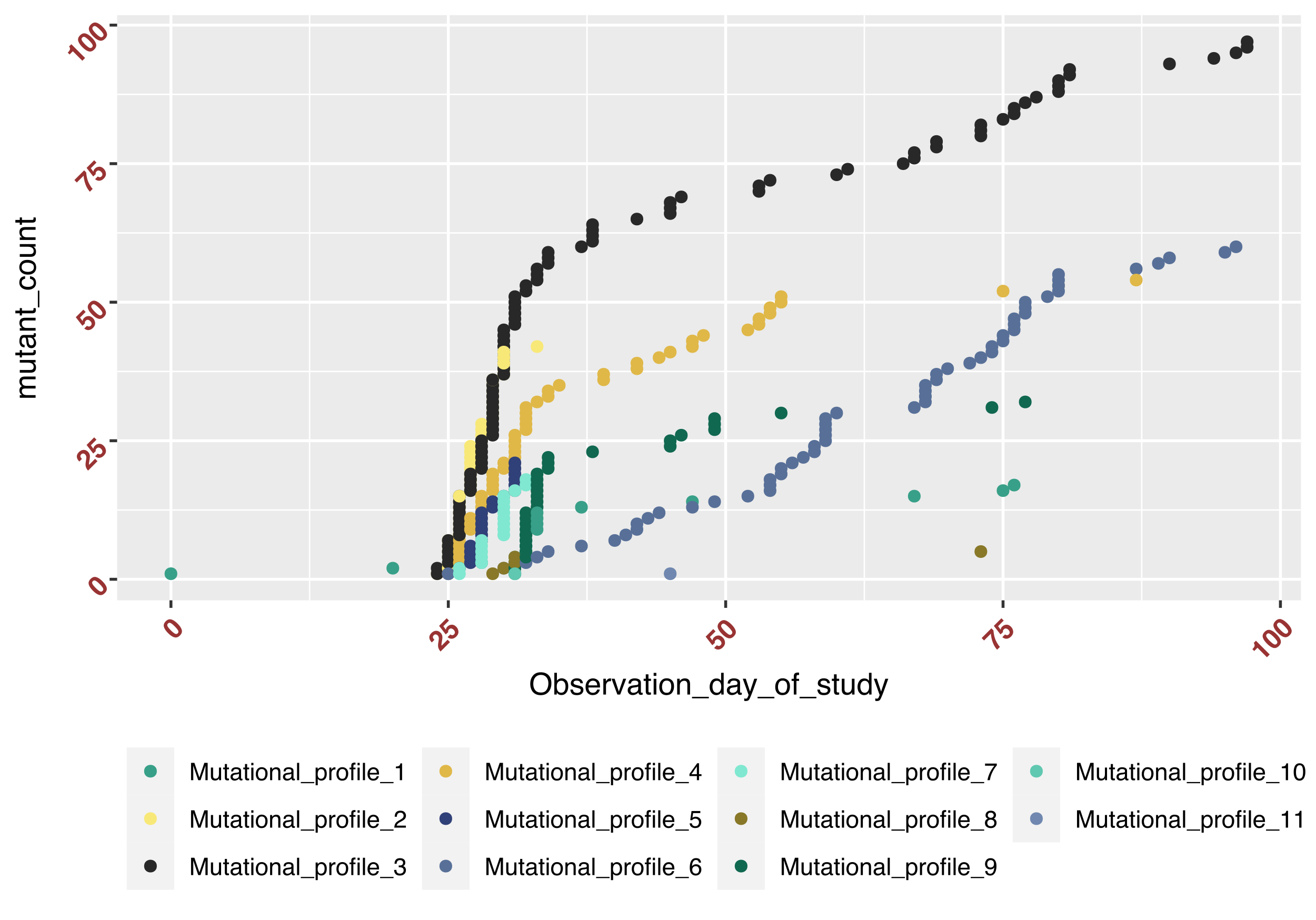

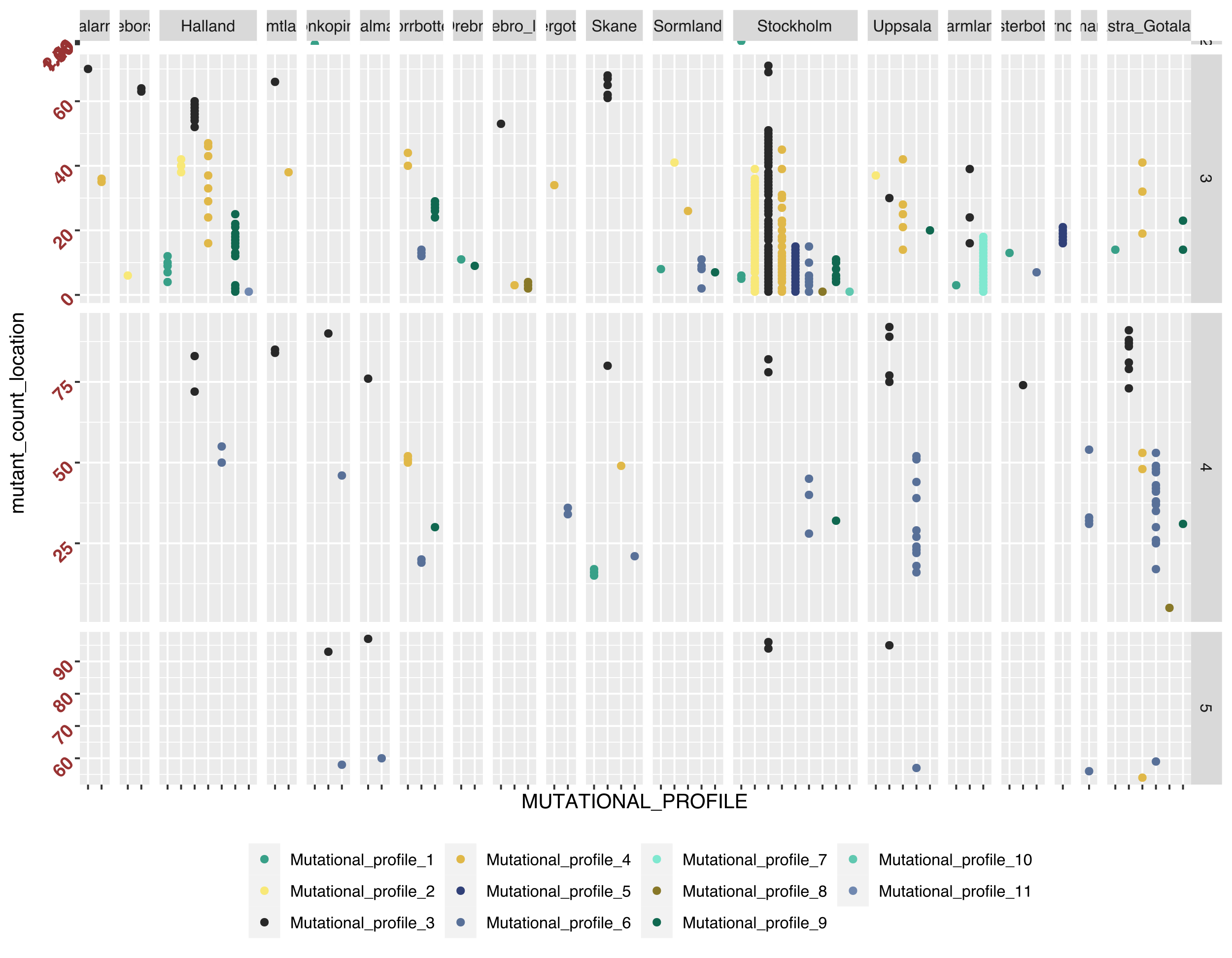


(**a**)

(**b**)
