# Supplementary material for: Spatio-Temporal Mutational Profile Appearances of Swedish SARS-CoV-2 during the Early Pandemic
Source: Viruses. 2020 Sep 14;12(9):1026. doi: 10.3390/v12091026 (PMC7551444; doi:10.3390/v12091026)
Supplement: Supplementary file 1 [file viruses-12-01026-s001.zip › Submission_mutation_cov_200824_Table_S2.docx]

**Supplemental Table 2. Model comparison by BEAST analysis**

| Molecular clock model | Demographic model | Mutation rate | 95%HPD | Log marginal likelihood |  |
| --- | --- | --- | --- | --- | --- |
|  |  |  |  | PS | SS |
| uncorrelated | exponential | 1.6979E-3 | [1.4249E-3, 2.0009E-3] | -46340.9739 | -46346.6604 |
| uncorrelated | skyline | 1.7634E-3 | [1.3768E-3, 2.165E-3] | -46315.1017 | -46324.6097 |
| random | skyline | 1.5041E-3 | [1.2225E-3, 1.7907E-3] | -34079.9983 | -34081.8943 |
| random | exponential | 1.5914E-3 | [1.3335E-3, 1.8324E-3] | -42896.5206 | -42901.6690 |
| strick | skyline | 1.2328E-3 | [9.979E-4, 1.499E-3] | -46342.3776 | -46348.7706 |
| strick | exponential | 1.5425E-3 | [1.2795E-3, 1.8259E-3] | -46368.2393 | -46374.2749 |

95% HPD, 95% Highest Posterior Density; SS, step-stone sampling; PS, path sampling
