# Supplementary figures and images for: Spatio-Temporal Mutational Profile Appearances of Swedish SARS-CoV-2 during the Early Pandemic
Source: Viruses. 2020 Sep 14;12(9):1026. doi: 10.3390/v12091026 (PMC7551444; doi:10.3390/v12091026)

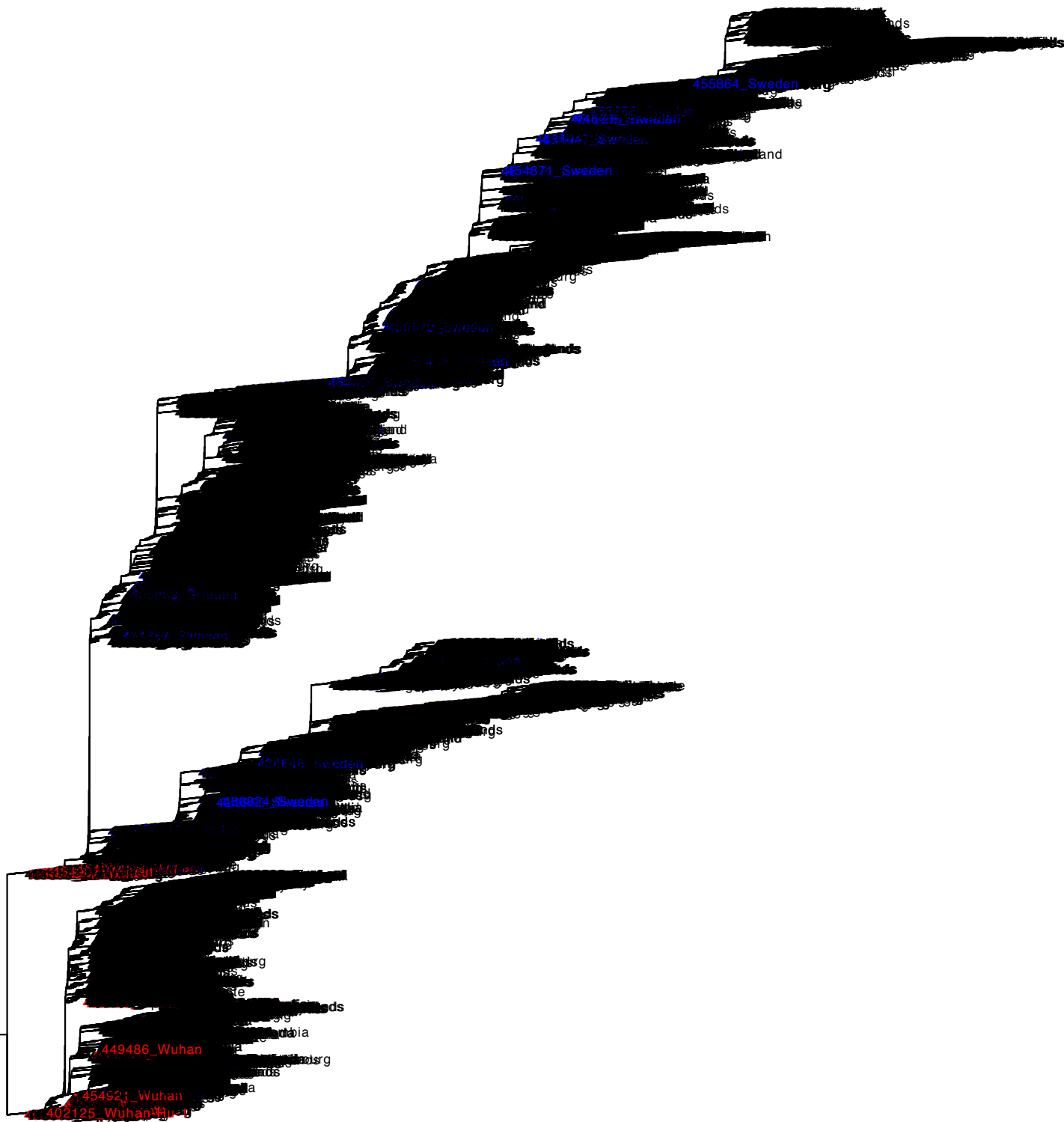

Supplement: Supplementary file 1 [file viruses-12-01026-s001.zip › Submission_mutation_cov_200824_Figure_S2.pdf]
